# The Burden of Non-Communicable Diseases (NCDs) among Prisoners in India: A Systematic Review and Meta-Analysis

**DOI:** 10.3390/healthcare10102046

**Published:** 2022-10-17

**Authors:** Subhanwita Manna, Snehasish Tripathy, Rahul Kumar Sah, Bijaya Kumar Padhi, Sandeep Kaur, Behdin Nowrouzi-Kia, Vijay Kumar Chattu

**Affiliations:** 1 Indian Institute of Public Health, Delhi 122002, India; 2 Department of Preventive Oncology, Homi Bhabha Cancer Hospital & Research Centre, Tata Memorial Hospital, Muzaffarpur 842004, India; 3 Asian Institute of Public Health, Bhubaneswar 754001, India; 4 Department of Community Medicine and School of Public Health, Postgraduate Institute of Medical Education and Research, Chandigarh 160012, India; 5 Department of Economic Studies, Central University of Punjab, Bathinda 151401, India; 6 ReSTORE Lab, Department of Occupational Science & Occupational Therapy, Temerty Faculty of Medicine, University of Toronto, Toronto, ON M5G1V7, Canada; 7 Center for Transdisciplinary Research, Saveetha Dental College, Saveetha Institute of Medical and Technical Sciences, Saveetha University, Chennai 600077, India; 8 Department of Community Medicine, Faculty of Medicine, Datta Meghe Institute of Medical Sciences, Wardha 442107, India

**Keywords:** noncommunicable diseases (NCDs), prevalence, prisoners, prisoners health, mental health, prevention, systematic review

## Abstract

Background: The increasing prevalence and subsequent mortality due to non-communicable diseases (NCDs) among Indian prisoners are often ignored by policymakers. This systematic review and meta-analysis aim to analyze the rising burden of Noncommunicable Diseases in Indian prisons and estimate the pooled prevalence of depression among Indian prisoners. Methods: A total 9 studies were chosen in accordance with PRISMA guidelines that investigated the burden of NCDs in Indian prisons and were published between January 2010 and August 2022. Statistical analysis was performed in STATA Version 16 software, and the funnel plot was used to identify publication bias. Results: A total of 167 articles were identified, and 9 were included in this analysis. The pooled prevalence of depression among prisoners was 48.78% (95% CI, 27.24–70.55%). According to the review, prisoners showed a significant prevalence of moderate to severe depression, dental caries, poor periodontal condition, and suicide ideation. This study is the first to analyze NCDs prevalence among Indian prisoners. Poor mental and dental health standards and the virtual absence of healthcare facilities necessitate governmental actions to boost inmates’ health. It is essential to develop preventative interventions for this extremely isolated and vulnerable group in addition to diagnosing and treating noncommunicable diseases. Conclusions: Our study findings will enable decision-makers to structure and develop appropriate preventative and curative programs for inmates’ general wellbeing.

## 1. Introduction

A “prison” is any jail or place where inmates are kept permanently or temporarily on the state government’s special or general instructions, including all lands and structures that are connected to it. It does not, however, include any places designated as subsidiary jails or used to imprison inmates who are just under the police’s care [1]. Prisoners are disproportionately comprised of members of the most underprivileged member of society with poor health and untreated chronic illnesses. This specific group of society is a neglected part of society. Their health issues are rarely addressed. They are significantly more susceptible to illness than the rest of the population because their health status is influenced by both their surroundings and the prisons in which they are incarcerated. At the end of 2021, there were 1306 prisons in India, with a 113.8% occupancy rate [2]. However, 44.1% of inmates are between the ages of 18 to 30, while 42.9% are between the ages of 30–50. In 2020, 1642 natural deaths were reported in the prisons of India, out of which cardiovascular diseases (CVDs) accounting 31.1%, followed by 14.5% for lung ailments and 4.2% for various types of cancer [3]. To ensure that prison health services satisfy the needs of their prisoners, accurate estimates of the prevalence of chronic diseases must be obtained among the prisoner population as it continues to grow, along with the number of elderly inmates. It is well-recognized that chronic infectious diseases are more common in jails. Still, less is known about the prevalence of chronic non-communicable diseases (NCDs) among inmates, particularly in the context of India. 

According to the WHO, the four major NCDs are diabetes, malignancies, chronic respiratory diseases, and cardiovascular diseases. NCD-related mortality accounts for around 3/4 of the global population, or 31.4 million people [4]. Non-communicable diseases (NCDs) are responsible for 60% of all mortality in India [5]. Even though NCDs impact people from all socioeconomic backgrounds, there are clear differences in the burden of NCDs, with those who are more vulnerable and have lower socioeconomic status were adversely affected [6].

There are no incidence or prevalence studies on NCDs in the prisoner population in India, and the available research appears to be limited to local descriptive health needs assessments. As a result, due to the dearth of scientific knowledge on the prevalence of NCDs in this vulnerable population, we conducted this analysis to assess the burden of non-communicable diseases among society’s most marginalized groups as well as the approaches to deal with the same.

## 2. Materials and Methods

### 2.1. Search Strategies

Four electronic databases (PubMed, Google Scholar, Web of Science, and Scopus) were searched for publications published between 1 January 2010, and 1 January 2022. Initially, 376 articles from PubMed, 2460 articles from Google Scholar, 92 from Scopus, and 135 from Web of Science were discovered; however, 3040 items were reviewed, and after the exclusion of publications based on title and abstract, a comprehensive review of 167 articles were conducted, with 9 papers being selected for review. 

The search terms included “NCDs,” “psychiatric disorders”, “cardiovascular diseases”, “Anaemia”, “Chronic respiratory illnesses”, “Oral disorders”, “Cancer” and “Musculo Skeletal disorder”, along with “prisoners”, “inmates”, and “India”. The essential phrases were used both separately and in conjunction with Boolean operators “AND” and “OR”. To find relevant publications on the study’s title, MeSH terms with an asterisk were applied (Table A1). To handle citations and expedite the review process, articles were downloaded to Zotero. We looked for papers that were published between 2010 and 2022.

### 2.2. Data Extraction and Management

**Figure 1:**
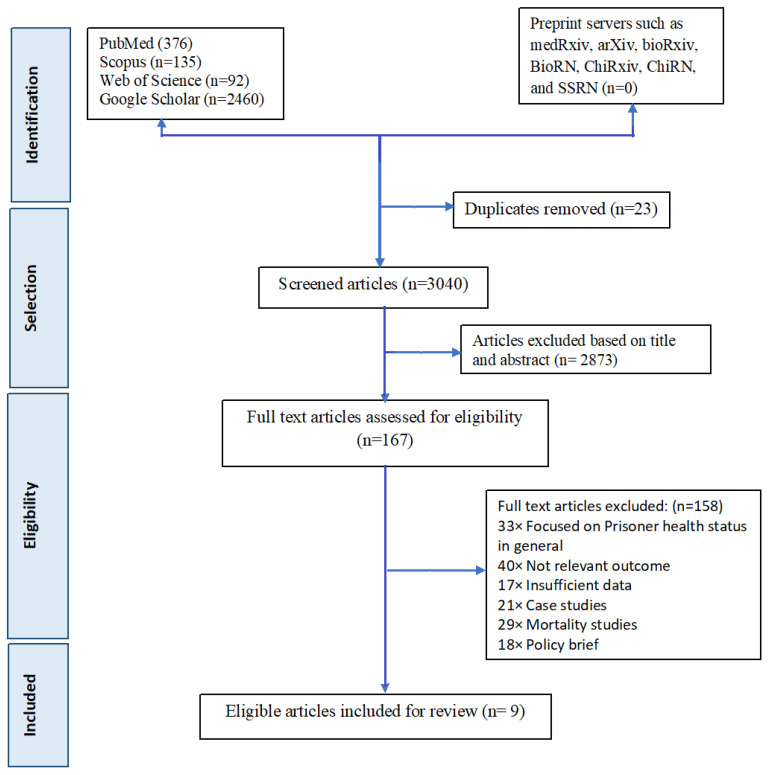
PRISMA flowchart for included studies in systematic review and meta-analysis of prevalence of NCDs among prisoners of India.

Two authors independently searched the papers. If there was a disagreement over the choice of an article, two of the co-authors discussed it and reached an agreement. If the two lead reviewers were discordant about the article’s eligibility, a third co-author was consulted to thoroughly assess the article and help to decide about the inclusion of the study. Reviewers ultimately discovered 9 articles that were relevant to the main topic. To maintain scientific accuracy in reporting searched publications, the Preferred Reporting Standard of Systematic Reviews and Meta-Analysis (PRISMA) checklist was followed (Figure 1). The reviewers attentively read these 9 publications before tabulating their results.

### 2.3. Inclusion and Exclusion Criteria

The COCOPOP (Condition, Context, and Population) paradigm was used to examine the eligibility of the included publications in this systematic review and meta-analysis [7]. Prisoners were the study population (POP), the condition (CO) was the prevalence of NCDs, and the context (CO) was only Indian studies. The lists of precise inclusion and exclusion criteria are available in Table A2.

### 2.4. Quality Assessment

Two (2) authors independently assessed the studies using the NHLBI-Quality assessment tool [8]. Table A3 displays the results for all investigations across all fourteen domains.

### 2.5. Statistical Analysis

By dividing the number of positive individuals by the total number of study participants, the prevalence of mental health disorders (Moderate-Severe depression) was determined. The I2 test was used to evaluate the heterogeneity of the studies used in this meta-analysis. The degree of variation between research is referred to as heterogeneity. According to the I2 values of less than 25%, 25–50%, and more than 50%, the studies’ heterogeneity was categorized as low, moderate, and high, respectively [9]. A significant amount of heterogeneity among the papers made up the meta-analysis. Therefore, a random-effect model with a 95% confidence interval was utilized to estimate the overall effect. *p* < 0.05 was regarded as statistically significant when performing the meta-analysis using STATA software (version 16, STATA Corp).

## 3. Results

This review covered 2676 prisoners from various Indian central and district level prisons. Table 1 shows the baseline characteristics of studies examining NCD among prisons in India. Most of the prisoners were male and belonged to the age group of 18–96 years. However, the socio-economic status of the majority of the prisoners belongs to the low-middle income group. 

### 3.1. Mental Health Status in the Prisons

**Figure 2:**
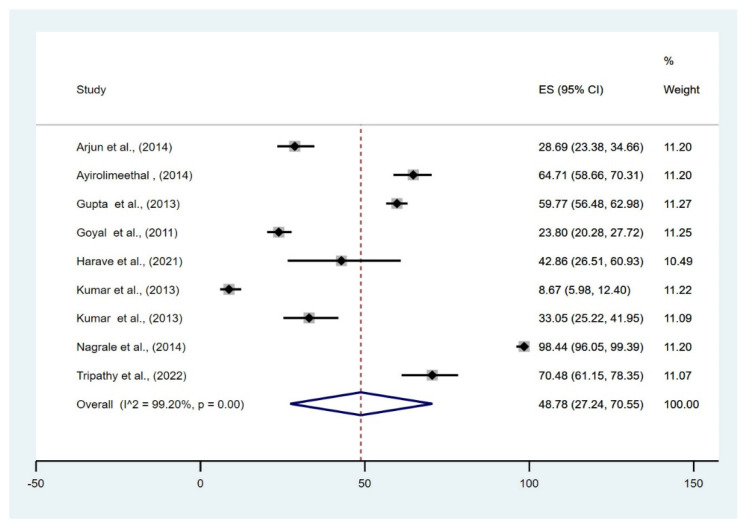
Forest plot of pooled magnitude of NCDs among prisoners of India [1,10,11,12,13,14,15,16,17].

**Figure 3:**
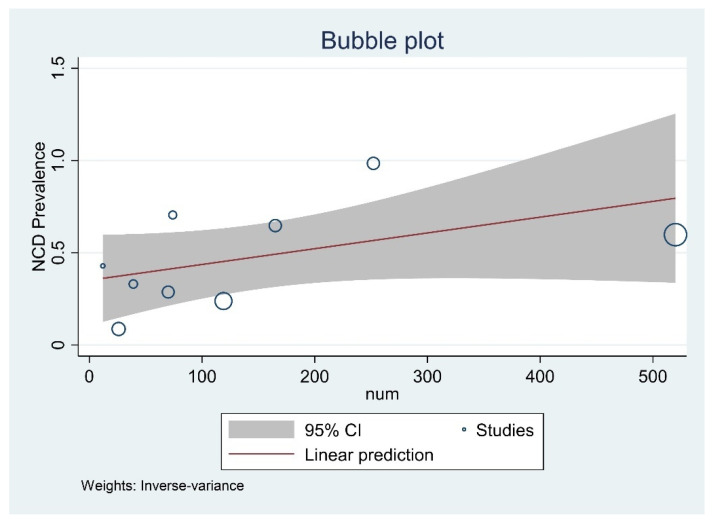
Bubble plot with 95% Confidence interval of pooled prevalence of NCDs among prisoners of India.

Six studies evaluated the prisoners’ mental health. The meta-analysis of the moderate-severe depression data reported that the pooled prevalence among prisoners was 48.78% (95% CI, 27.24–70.55%) across all district and central prisons of India. Since there was no heterogenicity included in the study, a fixed model was used (I2 = 99.20%, *p* = 0.001) (Figure 2). The bubble plot depicted that the studies that reported a higher prevalence of depression tend to have a larger sample size (Figure 3). 

The majority of the research papers reported that depression is the most common psychiatric disorder among inmates. For instance, a study reported that 23.8% of the imprisoned people at Central Jail in Amritsar had psychiatric illnesses other than substance misuse [13]. Among the prisoners, depression, schizophrenia-like symptoms, and suicidal ideation were the most often seen mental health issues. Inmates of an Odisha jail were found to have severe to moderate depression in 53.3% of cases, according to a study by Tripathy et al. [10]. According to this study, the educational status and level of social support from family members and other jail prisoners were the major determinants of depression behind bars. A similar study found that 33% of offenders in Kota’s major jail had psychiatric problems overall, with 6.7% having psychotic disorders, 16.1% having depressive disorders, and 8.5% having anxiety disorders [11]. Another study conducted in the district jail in Kozhikode, Kerala, discovered that 6.3% of prisoners had psychosis, 13.7% had adjustment issues, 4.3% had mood disorders, and 19.2% had antisocial personality disorder. A significant percentage of male prisoners (69.7%) were found to have a current mental health issue [12].

Another study, however, found that 0.67% of prisoners displayed signs of schizophrenia, while 1% of prisoners had severe depression and seizures [1]. This finding, supported by another study done in the Central Jail of Belgaum, represents that the majority of the prisoners had low depression; however, the prevalence of moderate to severe depression was 4.85%. In the early stages of conviction, depression (57.13%) was substantially more prevalent. Inmates who had been incarcerated for more than six years had a prevalence of 42.85%. In contrast, those who had been incarcerated for less than six years had a quite high prevalence of 57.13%, indicating that the length of the prison sentence was negatively connected with depression [14].

### 3.2. Publication Bias 

Egger’s test for a regression intercept gave a *p*-value of 0.974, indicating no evidence of publication bias.

### 3.3. Prevalence of Oral Disease 

4 studies assessed the oral health status of the inmates. Most of the studies reported that dental caries is the most common type of oral health issue faced by prisoners, followed by oral mucosal lesions and dental fluorosis. A study revealed a similar finding carried out in a Karnataka prison that 98.5% of people had periodontal disease, and 82.42% had dental caries. Most inmates (98%) did not receive any form of dental care while incarcerated. The study also relates prisoners’ dental issues to unhealthy habits, including smoking and the use of smokeless tobacco [18]. Another study conducted in the central jail of Bhopal among both the psychiatric and non-psychiatric inmates reported that the overall prevalence of oral lesions was 34.8%, comprising psychiatric inmates (39.3%) and non-psychiatric inmates (30.3%) [16]. 

Most of the studies depicted that majority of the prisoners have poor oral health, for instance, one of the studies conducted in the District Jail of Mathura reported that 79% of the prisoners had a poor periodontal condition and dental caries, followed by 59.8% inmates had pro-mucosal lesions. However, this study also reported that all the inmates demonstrated signs of dental fluorosis; 58.6% had mild, 27.8% had moderate, and 4.3% had severe fluorosis [15]. On the contrary, another study conducted in the Central Jail of Gulbarga city reported that only 7.33% of inmates had dental caries among all the prisoners [1].

### 3.4. Other NCDs

We did not find many studies that surveyed the prisoners’ major NCD status. We found just a single report which revealed that among the prisoners, the prevalence of anaemia, diabetes mellitus, epilepsy, presbyopia, senile cataract and myopia, acute conjunctivitis, conductive deafness, otitis media, and circulatory system diseases was 84%, 2.33%, 1%, 3.67%, 2.67%,0.67%, 1.67%, 0.67% and 4%, respectively [1]. 

## 4. Discussion

Most research on the prevalence of NCDs and the treatment needs of prisoners has been conducted in Western countries. A thorough search of the literature revealed studies on the health status of prisoners in India. Still, the majority of these studies focused on the knowledge assessment of prisoners’ general health status, and very few studies were conducted on the burden of NCDs among prisoners, indicating that their health status is frequently neglected. Due to the dearth of available data, the present study aimed to evaluate the NCD burden among Indian inmates. This review of 9 studies provides a partial overview of the burden of NCDs among the inmates as a smaller number of studies addresses the issues of NCDs among the most neglected population of society. According to our findings, depression is the most common type of mental condition among prisoners. The main factors influencing this are a lack of social support and substance misuse. This finding is supported by another systematic review conducted in LMICs found that the estimated pooled prevalence of major depression among prisoners was 16% (11.7–20.8), which is considerably higher than the general population [19]. 

The study reported that the pooled prevalence among prisoners was 48.78% (95% CI, 27.24–70.55%) which is in line with another meta-analysis conducted in the prison of Ethiopia reported that the pooled prevalence of depression among Ethiopian prisoners was 44.45 (95% CI: 40.28, 48.61). The depression prevalence among Nepali prisoners also reported nearly similar results [20]. Our study also reported a low prevalence of schizophrenia among the prisoners, which is aligned with another study by Falissard et al., who found that 3.8% of French inmates had schizophrenia and 17.9% had major depression. In developing nations like India, the prevalence of mental diseases is low compared to Western nations [21].

A high proportion of significant mental illness among Indian prisoners may be attributed to the country’s poor community mental health-care facilities, which have yet to reach its socially disadvantaged and marginalized citizens. According to reports, low-resource environments have a higher occurrence of human rights violations involving detained people with mental health disorders, particularly those with psychotic illnesses [22].

This present study finds that among all oral problems, the prevalence of dental caries is more common among prisoners. These findings are in agreement with a multicentered oral health survey carried out in 2007–2008 in collaboration with WHO India and the Government of India’s Department of Health, which discovered that the prevalence of dental caries ranged from 23.0% to 71.5% in 12-year-old and from 48.1% to 86.4% in adults aged 35–45 years. On the other hand, the prevalence of dental caries among older adults aged 65 to 74 years ranged from 51.6–95.1%. The occurrence of periodontal disease in adults ranged from 15.32% to 77.9%, while in the aging population, it ranged from 19.9% to 96.1% [22]. However, Kumar et al. conducted a review that reported that, irrespective of their age and gender, most prisoners have periodontal disease and tooth decay. The study also concluded that the prison population exhibited a higher risk of tooth cavities and poor periodontal health than the general population [23]. 

This review finds that the anaemia prevalence among the prisoners was quite high compared to the general population, this finding is supported by a study conducted by Lokpo et al., which reported anaemia prevalence among the prisoners of Ho Central Prisons was 31.86% [24]. 

### 4.1. Recommendations

The present review yields several recommendations. Early detection, appropriate management, and prompt follow-ups are essential for the efficient monitoring and management of health conditions among prisoners. Delayed diagnosis and recently found outcomes may lead to increased morbidity, mortality, and the burden of the diseases. Due to barriers brought on by substance abuse and a lack of active follow-up, some people run the risk of not obtaining services even when they are diagnosed with an illness in time for treatment. Therefore, a pragmatic intervention program can be developed to provide preventive and curative care to prisoners. However, it is challenging for prisoners to obtain timely and effective medical care since most jails, especially those in developing countries, are understaffed and under-resourced.

Additionally, Indian prisoners are not well-aware of the need for medical care. The complexities in obtaining authorization to do medical examinations, treatments, and diagnostic tests inside prisons highlight the need for additional public health research in India. More data and research are required to determine the variables affecting the health status of the population in prison, not only in India but also in developing countries. Determining if incarceration or other systemic factors contribute more to the elevated risk of NCD is equally crucial. Furthermore, future research initiatives may include cross-sectional surveys to ascertain the prevalence and type of NCDs and risk factors related to them among prisoners. The appropriate timeframe for active case chasing among prisoners or follow-up screening after release should be determined. A follow-up study of the suggested service supply would also be valuable. It would also be beneficial to implement treatments to assess self-management support.

### 4.2. Limitations

One of the key limitations of this systematic review is the variation in the sample sizes of prisoners analyzed in the various studies. Even though we have included studies conducted in different regions of India, resulting in a geographically varied sample, it would have been assumed that the prevalence of oral health and mental health concerns would vary significantly due to differences in jail systems, access to counsellors, dentist, food habits, and data collection processes. Furthermore, the studies included in this review were all relied on observational data.

## 5. Conclusions

The epidemic has caused tremendous disruption in society, but it has also highlighted that “business as usual” falls short of meeting the unmet needs of disadvantaged people. This must be an opportunity to reset our society’s moral compass to ensure that historical injustices are mitigated, that everyone has access to health care, and that no one is left behind.

## Figures and Tables

**Table 1:** Baseline characteristics of studies examining NCD among Prisons in India (N = 9).

SL No	Author/YOP	Study Design	Sample	Prevalence	NCD Type	Age	Participants	
							Male	Female
1	Tripathy et al., 2022 [10]	Cross-sectional	105	27.6%	Depression	18–65	Male	
2	Kumar V et al., 2013 [11]	Cross-sectional	118	33%	Psychiatric disorders	19–66	Male	Female
3	Ayirolimeethal A et al., 2014 [12]	Cross-sectional	255	68.60%	Psychiatric disorders	18–78	Male	Female
4	Goyal SK et al., 2011 [13]	Cross-sectional	500	23.80%	Psychiatric disorders	NA	Male	Female
5	Harave S V et al., 2021 [14]	Cross-sectional	28	57.13%	Depression	30–60	Male	Female
6	Gupta R et al., 2013 [15]	Cross-sectional	870	59.80% 58.60% 21.30%	Oral Mucosal lesions Dental Fluorosis Dental caries	18–85	Male	Female
7	Arjun NT et al., 2014 [16]	Cross-sectional	244	54.2% 14.8% 12.8% 35.80%	Depression Schizophrenia Anxiety Disorder Oral mucosal lesions	34–96	Male	
8	Kumar D et al., 2013 [1]	Cross-sectional	300	18% 84% 4% 7.33%	Musculo skeletal disorder Anaemia Hypertension Dental Carries	20–50	Male	Female
9	Nagrale et al., 2014 [17]	Cross-sectional	256	98.50% 82%	Periodontal disease Dental Carries	18–27	Male	Female

## Data Availability

Data will be made available upon reasonable request to the corresponding author.

## Appendix A

**Table A1:** The adjusted search terms as per searched electronic databases.

Database	No	Search Query	Results
**PubMed**			
	#1	(((((((((((((((((((((((((((((((((((((((((((((((((((((NCD) OR (Non-communicable diseases)) OR (cancer)) OR (cardiovascular diseases)) OR (ischemic heart disease)) OR (neoplasm)) OR (mental health)) OR (depression)) OR (loneliness)) OR (oral health)) OR (periodontal status)) OR (oral cancer)) OR (diabetes)) OR (diabetes mellitus)) OR (anemia))) OR (chronic diseases)) OR (non-infectious diseases)) OR (hypertension)) OR (Atherosclerosis)) OR (stroke)) OR (Cerebrovascular Disorders)) OR (Myocardial Ischemia)) OR (Heart Failure)) OR (Heart Diseases)) OR (acute coronary syndrome)) OR (hypertensive heart disease)) OR (cardiac failure)) OR (myocardial infarction)) OR (HTN)) OR (vascular disease)) OR (double vessel disease)) OR (glucose tolerance)) OR (tumour)) OR (tumors)) OR (Neoplasms by Site)) OR (oral diseases)) OR (blood pressure)) OR (chronic kidney diseases)) OR (osteoarthritis)) OR (Alzheimer’s disease)) OR (cataracts)) OR (chronic lung illnesses)) OR (endocrine disorders)) OR (neuropsychiatric conditions)) OR (digestive diseases)) OR (rheumatoid arthritis)) OR (mental disorders)) OR (major depressive disorders)) OR (schizophrenia)) OR (anxiety disorders)) AND (India)))	244,684
	#2	((((((((((((((prisoners) OR (prison)) OR (jail)) OR (correctional settings)) OR (criminals)) OR (detention centre)) OR (gaol)) OR (detainee)) OR (incarceration)) OR (“correctional facilities” [All Fields])) OR (correctional settings)) OR (offender)) AND (India))))	1284
	#3	#1 AND #2	376
**Scopus**			
	#1	(TITLE-ABS-KEY (Non-communicable diseases) OR TITLE-ABS-KEY (NCD) OR TITLE-ABS-KEY (cardiovascular diseases) OR TITLE-ABS-KEY (cancer) OR TITLE-ABS-KEY (Diabetes)) OR TITLE-ABS-KEY (Chronic lung diseases))AND (India)))	
	#2	(TITLE-ABS-KEY (prison) OR TITLE-ABS-KEY (jail) OR TITLE-ABS-KEY (detainee) OR TITLE-ABS-KEY (incarceration) OR TITLE-ABS-KEY (Inmates)) AND (India)))	
	#3	#1 AND #2	135
**Web of Science**			
	#1	Non-communicable diseases (All Fields) or NCDs (All Fields) or cardiovascular diseases(All Fields) or cancer (All Fields) or Diabetes(All Fields) or Chronic lung diseases (All Fields) orAnemia (All Fields) or Psychiatric disorder (All Fields) or Oral Health (All Fields) AndIndia (All Fields)	
	#2	Prison (All Fields) or Inmates (All Fields) or Detainee (All Fields)or incarceration (All Fields) AndIndia (All Fields)	
	#3	#1 AND #2	92
**Google Scholar**		((((((((((((((prisoners) OR (prison)) OR (jail)) OR (correctional settings)) OR (criminals)) OR (detention centre)) OR (gaol)) OR (detainee)) OR (incarceration)) OR (“correctional facilities” [All Fields])) OR (correctional settings)) OR (offender)) AND (India))))	
	with at least one of the words	Non communicable disease	25,200
	with at least one of the words	Prisoners or inmate or jail or culprit or detainee or victim and India	15,700
	Final		2460

**Table A2:** Inclusion and exclusion criteria.

Inclusion		Exclusion
**Participants**	Male or Female Any length of time in prison Any age group	Prisoners of war Recently released/parole Young offenders Secure Psychiatric Units Prison population sub-groups
**Disease**	- Non-communicable disease as defined by the GBD study; - Cardiovascular diseases (including hypertension) - Neoplasms - Other non-communicable diseases (congenital, oral, skin, sensory) - Musculoskeletal disorders - Diabetes, urogenital, blood, and endocrine diseases - Chronic respiratory diseases - Neurological disorders - Oral health	Communicable disease Trauma/accidents Studies focused only Dental Carries Prevalence of risk factors for disease (such as obesity, hypercholesterolaemia, smoking, alcohol, drug use etc) Pre-cancerous change (CIN, CIS etc)
**Outcome**	Prevalence measured from routine data sources such as; prison databases, medical notes, prescription lists, physical examinations, diagnostic tests, survey or self-reported	Prison as a risk factor for a disease in a wider population
	Data is age-stratified or presented for those aged 50 years or older only	Mortality studies
**Study**	Prevalence studies, cross-sectional studies, cohort studies, case control studies, surveys	Qualitative, policy, opinion, case-studies, case-series studies
	India Published between 2010–2022 English Language	
	Published and Un-published data	

**Table A3:** Quality assessment of studies examining NCD among Prisons in India (N = 9) [1,10,11,12,13,14,15,16,17].

Authors/DOP	Tripathy et al., 2022 [10]	Kumar V et al., 2013 [11]	Ayirolimeethal A et al., 2014 [12]	Goyal SK et al., 2011 [13]	Harave S V et al., 2021 [14]	Dhanker k et al., 2013 [15]	Arjun NT et al., 2014 [16]	Kumar D et al., 2013 [1]	Nagrale et al., 2014 [17]
1. Was the research question or objective in this paper clearly stated?	Yes	Yes	Yes	Yes	Yes	Yes	Yes	Yes	Yes
2. Was the study population clearly specified and defined?	Yes	Yes	Yes	Yes	Yes	Yes	Yes	Yes	Yes
3. Was the participation rate of eligible persons at least 50%?	Yes	Yes	Yes		Yes	Yes	Yes	Yes	Yes
4. Were all the subjects selected or recruited from the same or similar populations (including the same time period)? Were inclusion and exclusion criteria for being in the study prespecified and applied uniformly to all participants?	Yes	Yes	Yes	Yes	Not Reported	Yes	Yes	Yes	Not Reported
5. Was a sample size justification, power description, or variance and effect estimates provided?	Yes	Yes	Not Reported	Not Reported	Not Reported	Not Reported	Not Reported	Not Reported	Not Reported
6. For the analyses in this paper, were the exposure(s) of interest measured prior to the outcome(s) being measured?	Not Applicable	Not Applicable	Not Applicable	Not Applicable	Not Applicable	Not Applicable	Not Applicable	Not Applicable	Not Applicable
7. Was the timeframe sufficient so that one could reasonably expect to see an association between exposure and outcome if it existed?	Cannot Determine	Cannot Determine	Cannot Determine	Cannot Determine	Cannot Determine	Cannot Determine	Not Reported	Not Reported	Cannot Determine
8. For exposures that can vary in amount or level, did the study examine different levels of the exposure as related to the outcome (e.g., categories of exposure, or exposure measured as continuous variable)?	Cannot Determine	Cannot Determine	Cannot Determine	Cannot Determine	Cannot Determine	Cannot Determine	Yes	Cannot Determine	Yes
9. Were the exposure measures (independent variables) clearly defined, valid, reliable, and implemented consistently across all study participants?	Yes	Yes	Yes	Yes	Yes	Yes	Yes	Yes	Yes
10. Was the exposure(s) assessed more than once over time?	Not Applicable	Not Applicable	Not Applicable	Not Applicable	Not Applicable	Not Applicable	Not Applicable	Not Applicable	Not Applicable
11. Were the outcome measures (dependent variables) clearly defined, valid, reliable, and implemented consistently across all study participants?	Yes	Yes	Yes	Yes	Yes	Yes	Yes	Yes	Yes
12. Were the outcome assessors blinded to the exposure status of participants?	Not Applicable	Not Applicable	Not Applicable	Not Applicable	Not Applicable	Not Applicable	Not Applicable	Not Applicable	Not Applicable
13. Was loss to follow-up after baseline 20% or less?	Not Applicable	Not Applicable	Not Applicable	Not Applicable	Not Applicable	Not Applicable	Not Applicable	Not Applicable	Not Applicable
14. Were key potential confounding variables measured and adjusted statistically for their impact on the relationship between exposure(s) and outcome(s)?	Yes	Yes	Yes	Yes	Yes	Yes	Yes	Yes	Yes
**Final remarks**	Good	Good	Fair	Fair	Fair	Fair	Good	Good	Fair
